# Fixation of an Anatomically Designed Cementless Stem in Total Hip Arthroplasty

**DOI:** 10.1155/2012/912058

**Published:** 2012-04-10

**Authors:** Shigeru Nakamura, Noriyuki Arai, Takateru Kobayashi, Takashi Matsushita

**Affiliations:** Department of Orthopaedic Surgery, Teikyo University School of Medicine, Kaga 2-11-1, Itabashi-Ku, Tokyo 173-8606, Japan

## Abstract

*Purpose*. The Anatomic Fiber Metal plus stem (Zimmer) is one of the anatomically designed cementless stems to achieve stable fixation by metaphyseal fit. We studied outcomes of cementless total hip arthroplasty using this stem and possible effects of metaphyseal fit on outcomes. *Methods*. The cementless total hip arthroplasty using this stem was performed for 155 hips. One hundred and thirty-seven hips of 122 patients were followed for 5 to 16 (mean, 9.7) years and entered into the study. The metaphyseal fit was defined as good or poor in an anteroposterior radiograph after surgery. We studied the fixation of the stem and bone reaction on an anteroposterior radiograph at the final followup. *Results*. Twelve hips had revision, six acetabular components and six acetabular liners. No stem was revised. The biological fixation of the stem was bone ingrown fixation for 136 hips and unstable for one. The metaphyseal fit was good for 83 hips and poor for 54 hips. There were no differences for stem fixation and bone reaction between the two groups. *Conclusions*. The fixation of the stem was stable at a mean followup of 9.7 years independently from metaphyseal fit.

## 1. Introduction

**Figure 1:**
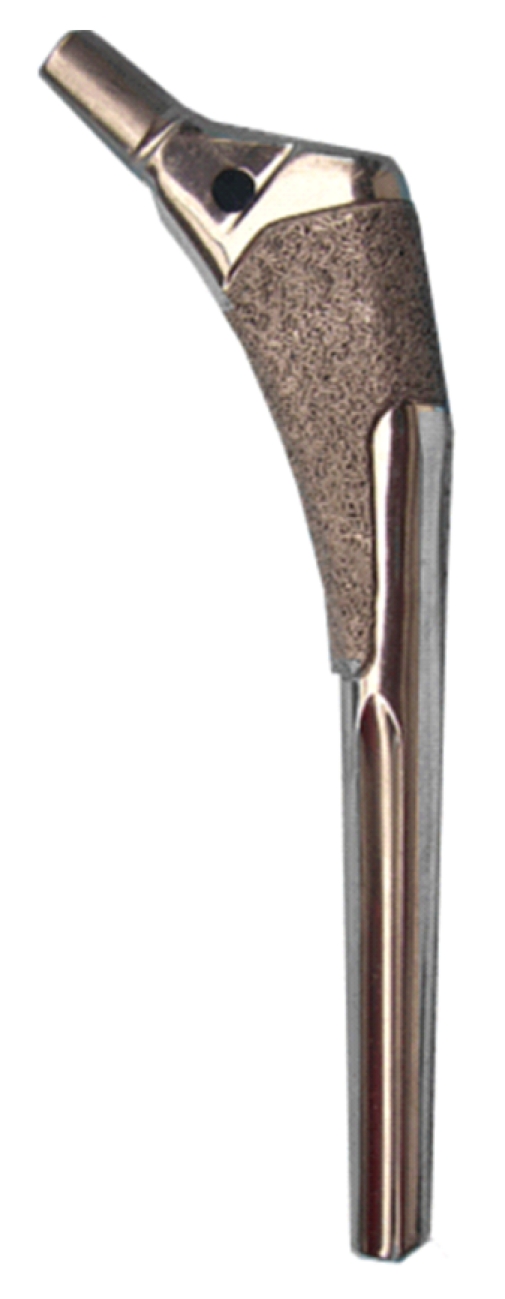
The Anatomic Fiber Metal plus stem.

For cementless total hip arthroplasty (THA), a large variety of femoral component designs have been developed. The Anatomic Fiber Metal plus stem (Zimmer, Indiana, USA) is one of the anatomically designed femoral components to be inserted without cement (Figure 1). The concept of this stem was to achieve stable fixation by metaphyseal fit and fill [1, 2]. It has a configuration matching a medullar canal of a normal femur and circumferential fiber-mesh coating on the proximal one-third. The neck of the stem has an anteversion of twelve degrees.

The press-fit and outcomes of THA using this stem were reported to be good for the primary osteoarthritis in selected Caucasian patients [1]. However, there were a few reports on the outcomes of THA using this stem in Japanese patients. The majority of the hips with osteoarthritis are dysplastic hips in Japanese patients [3]. Therefore, the results might be different from those in Caucasian patients.

We studied outcomes of cementless total hip arthroplasty (THA) using the Anatomic Fiber Metal plus stem in Japanese patients and possible effects of metaphyseal fit on outcomes.

## 2. Methods

The cementless total hip arthroplasty using the Anatomic Fiber Metal plus stem was performed for 155 hips of 139 patients between February 1994 and August 2003 at our hospital. Eighteen hips of 17 patients were excluded for the following reasons. Six patients (seven hips) had died during followup, eight patients could not be contacted, and the remaining three patients were contacted by telephone and confirmed to have no revision and to have no hip pain, but did not visit our clinic. One hundred and thirty-seven hips of 122 patients were followed for more than five years and entered into the study of clinical and radiographic outcomes.

The average follow-up period of the study group was 9.7 (5–16) years, and the average age at the surgery was 62 (33–80) years old. The diagnosis was osteoarthritis for 117 hips, osteonecrosis of the femoral head for 18 hips, and rapidly destructive coxarthrosis for two hips.

The indication of the usage of the Anatomic Fiber Metal plus stem was different according to the periods of the surgery. This stem had been used principally for all hips between February 1994 and May 1999 (defined as nonselection period). Between June 1999 and August 2003 (defined as selection period), we had used this stem as a first choice, but selected other stems (straight taper type or modular type) when the Anatomic Fiber Metal plus stem was not fit to the shape of medullar canal in an anteroposterior (AP) radiograph. During this period, we used the Anatomic Fiber Metal plus stem for 48% of all THA cases. Of the 155 hips inserted with this stem, 62 hips were operated in the nonselection period and 93 hips in the selection period.

The acetabular components were cementless spherical cups: HGP-II (Zimmer) for 22 hips and Trilogy (Zimmer) for 115 hips. The modular head was made of cobalt chromium alloy. The polyethylene of the acetabular liner was conventional for 51 hips and cross-linked for 76 hips.

**Figure 2:**
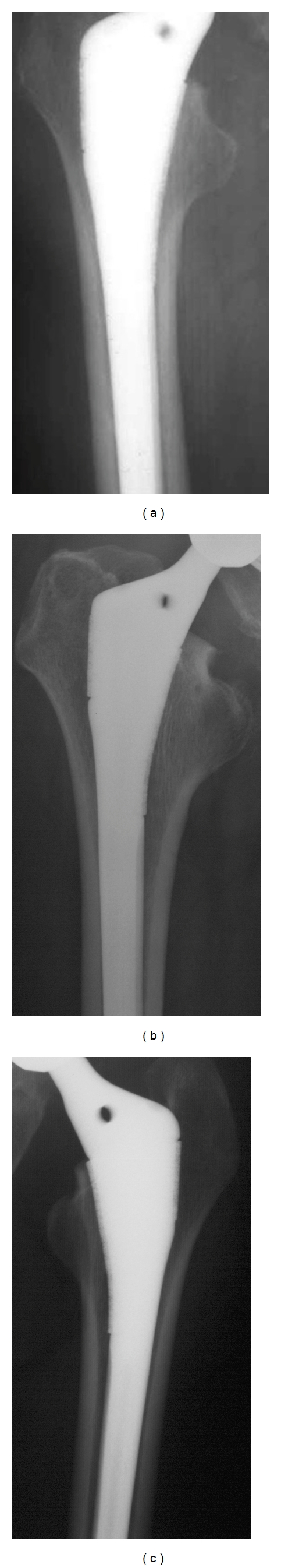
The metaphyseal fit evaluation in AP radiographs. (a) The medial side of the stem was in contact with the endosteum of the medial femoral cortex through the area of proximal fiber-mesh coating. The metaphyseal fit was defined as good. (b) The medial side of the stem was not in contact with the endosteum of the medial femoral cortex at any area of proximal fiber-mesh coating. The metaphyseal fit was defined as poor. (c) The medial side of the stem was not in contact with the endosteum of the medial femoral cortex at the distal area of proximal fiber-mesh coating. The metaphyseal fit was defined as poor.

We evaluated the metaphyseal fit on the postoperative AP radiograph and divided all hips into two groups (Figure 2). The metaphyseal fit was defined as good, if the medial side of the stem was in contact with the endosteum of the medial femoral cortex through the area of proximal fiber-mesh coating. The metaphyseal fit was defined as poor, if the medial side of the stem was not in contact with the endosteum of the medial femoral cortex at any point in the area of proximal fiber-mesh coating. We calculated the canal-filling ratio (CFR) at the distal end of the lesser trochanter and at the distal end of the stem in the poor metaphyseal fit cases to evaluate the stem size.

We studied the fixation of the components and bone reaction on an AP radiograph at the final followup. The biological fixation of the stem was classified into bone ingrown fixation, stable fibrous fixation, or unstable according to the methods of Engh et al. [4]. Unstable was defined as loosening. The subsidence of the stem more than four mm was defined as significant. The acetabular component having clear zone of more than 1 mm in all of the three zones of DeLee and Charnley [5] around the cup or change of inclination angle of more than 4 degrees was defined as loosening. The stress shielding was classified into four degrees according to the method of Engh et al. [4]. Radiolucent line, spot welds, and osteolysis were evaluated in seven zones of Gruen et al. [6] in AP radiographs.

The function of the hip was evaluated using the Japanese Orthopedic Association (JOA) hip score [7], with a full score of 100 points (pain 40, gait 20, range of motion 20, and activity of daily living 20 points).

We studied the revision rates and survival rates of all 155 hips using Kaplan-Meier methods. Chi-square test or Fisher test was used for categorical data, and Mann-Whitney test was used for numerical data. *P* value less than 0.05 was defined as significant.

This study was approved by the ethics committee of our institute and had been performed in accordance with the ethical standards laid down in the 1964 Declaration of Helsinki.

## 3. Results

Twelve hips including one hip with late infection had revision. The mean duration between total hip arthroplasty and revision was nine (1–16) years. No stem was revised. Six hips had revision of acetabular components, and the remaining six hips had revision of acetabular liners. For all 12 hips, conventional polyethylene liners had been used. Out of six acetabular revisions, three cups were well fixed, and the other three had no bony fixation. Well-fixed three cups were HGP-II cups. Cross-linked polyethylene liners were not available for HGP-II cups. We revised these cups to use cross-linked polyethylene liners. The reasons for liner revision were liner wear for three hips, late infection for one, dislocation for one, and dislodge of liner for one. For one hip of liner revision, bone graft was performed to osteolysis at the zone 1 of the femur.

The average JOA score of the study group was  44 ± 12  points before surgery and  87 ± 11  points at the final followup. One hundred three hips (75%) showed more than 80 points at the followup. Three hips had thigh pain.

**Figure 3:**
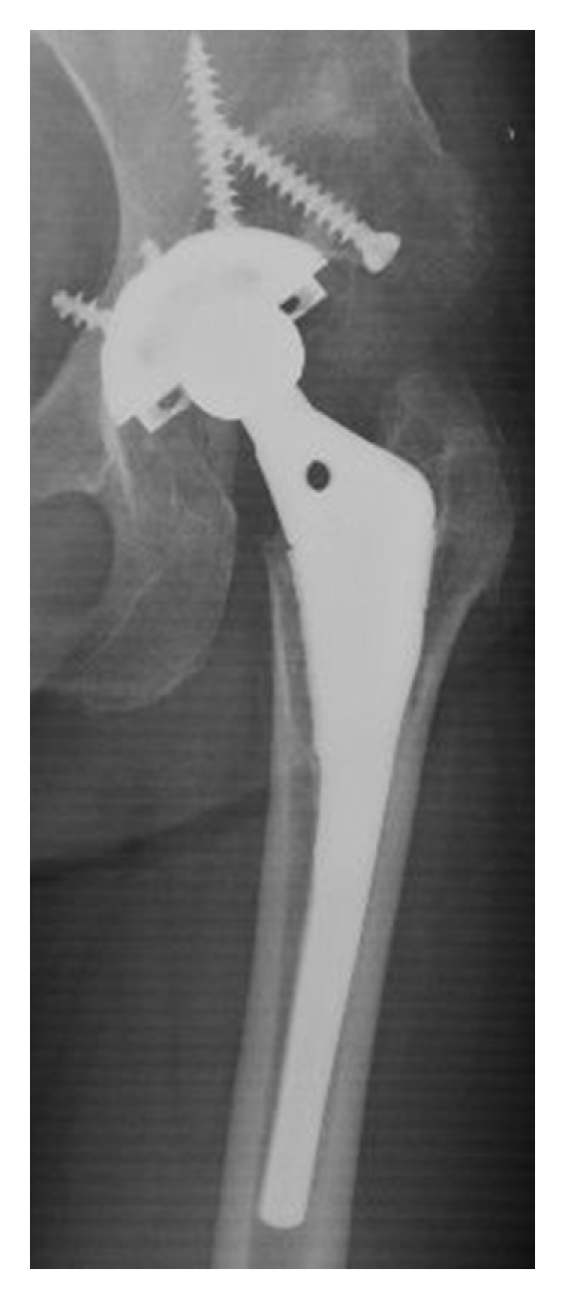
The AP radiograph of the left hip at 15 years after THA. The biological fixation of the stem was bone ingrown fixation.

**Figure 4:**
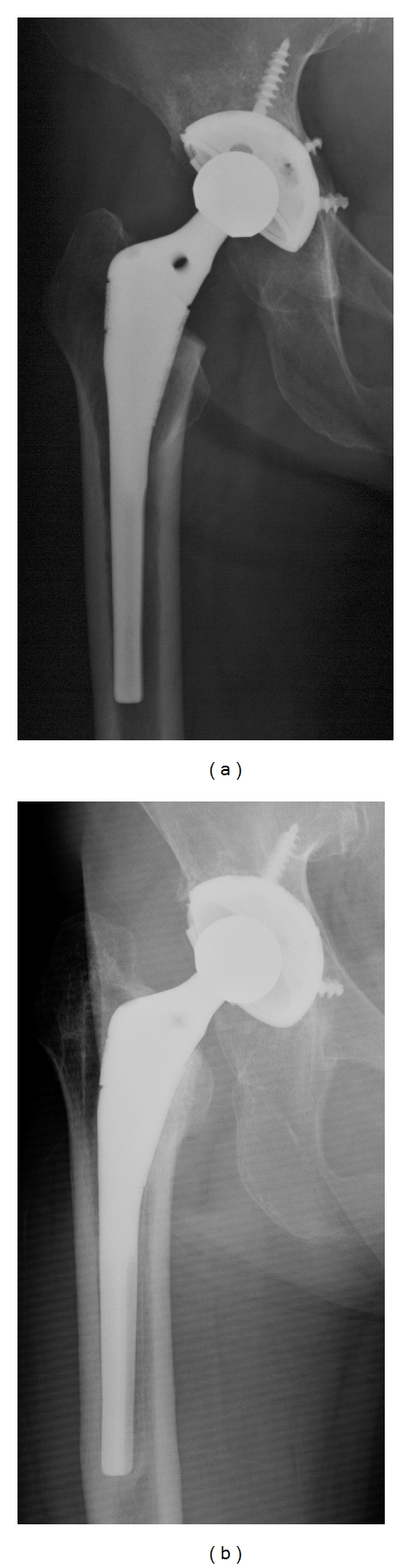
The right hip of 45-year-old female who had received bilateral THA for rapidly destructive coxarthrosis. (a) The postoperative AP radiograph. The stem had been undersized, and metaphyseal fit had been poor. (b) The AP radiograph at 6.1 years after THA. The stem showed subsidence and loosening.

The biological fixation of the stem was bone ingrown fixation for 136 hips (Figure 3) and unstable for one. The hip with unstable stem was the right hip of 45-year-old female who had received bilateral THA for rapidly destructive coxarthrosis. The metaphyseal fit had been poor on the postoperative AP radiograph (Figure 4). The stem had been undersized: CFR had been 0.63 at the distal end of the lesser trochanter and was 0.59 at the distal end of the stem. The follow-up radiographs showed no subsidence of the stem at three months after surgery, but subsidence of 5 mm at four years after surgery. The final follow-up radiographs at 6.1 years after surgery showed stem loosening with subsidence of 16 mm. She had died due to pulmonary disease not related to the hip before revision was performed.

Two hips showed subsidence. One hip was in the patient described above. The other hip had had femoral neck fracture during surgery. The stem had subsided 30 mm at six months after surgery, but showed no additional subsidence. At 7.5 years after surgery, the radiographs showed bone ingrown fixation. Ten-year survival rate was 94 (86–97)% when any surgery or revision for any reason was defined as end-point and was 99 (95–99.9)% when loosening or revision of the stem was defined as end-point.

Radiolucent lines of more than one mm were found at zone 1, 2, 5, and 6 of one hip with stem loosening (Figure 4(b)). Radiolucent lines of less than one mm were found at zone 2 of 6 hips, at zone 3 of 19 hips, at zone 4 of 106 hips (most frequent), at zone 5 of 46 hips, at zone 6 of 2 hips, and at zone 7 of one hip. No hip showed radiolucent lines of less than one mm at more than four zones. Spot welds were found at zone 6 of 108 hips. No spot welds were found at any other zones. Osteolysis was found at the medial side of greater trochanter in 18 hips (13%) and at zone 1 in one hip. No osteolysis was found at any other zone. Stress shielding was grade I for 133 hips and grade II for four hips.

Metaphyseal fit was good for 83 hips (61%) and poor for 54 hips (39%). In 54 hips with poor metaphyseal fit, mean CFR was 0.77 ± 0.07 (range, 0.59–0.92) at the distal end of the lesser trochanter and was 0.84 ± 0.09 (0.59–0.98) at the distal end of the stem. The CFR was less than 0.7 at both levels in only one hip shown in Figure 4(a). Other hips with low CRF at the distal end of the lesser trochanter showed good CFR at distal stem like the hip of Figure 2(b). The percentage of good was significantly higher in the selection period than in the nonselection period (69% versus 47%, *P* = 0.01). In diagnosis, the percentage of good was 59% for osteoarthritis and 78% for osteonecrosis. The hips with osteoarthritis showed a tendency of lower percentage of good, but no significant difference was found statistically (*P* = 0.13). We studied possible relationships between metaphyseal fit and outcomes of THA (Table 1). The duration of followup showed no differences between the good group and the poor group. There were no differences for JOA score at the followup, stem fixation, the rate of positive radiolucent line in zone 4, spot welds in zone 6, osteolysis at medial side of the greater trochanter, and stress shielding between two groups.

## 4. Discussion

Several studies [1, 2, 8, 9] on the outcome of THA using the Anatomic stem (Zimmer, Indiana, USA) in Caucasian patients reported that the rates of stem revision due to loosening were low (from 0 to 2.6%). There were two reports on the outcomes in Japanese patients. Harada et al. [10] reported that five cups and no stem had been revised in 81 hips with a mean followup of 8.4 years. Nakoshi et al. [11] also reported that four cups and no stem had been revised in 20 hips with a mean followup of 12.8 years. In our study, no stem had been revised and one stem showed loosening in 137 hips with a mean followup of 9.7 years. These results suggest that the biological fixation of this stem is good for 8 to 12 years after surgery not only in Caucasian but also in Japanese patients.

There was only one study that evaluated the metaphyseal fit or press-fit of the Anatomic stem. Ragab et al. [1] evaluated the press-fit of the stem in 97 hips using the methods of Callaghan et al. [12] and reported that the press-fit was excellent in 58 hips, good in 38 hips, and poor in one hip. These results suggest that the press-fit of this stem is good for the hip with primary osteoarthritis in Caucasian patients. However, direct comparisons to our results are not proper, because we had not used the evaluation methods of Callaghan et al. [12]. In their methods, the press-fit was defined as excellent if the AP radiograph showed the stem to be in contact with the cortical bone at some point on both the medial and the lateral surface. The Anatomic stem has no lateral flare to contact with the endosteum of the lateral metaphyseal cortex around the innominate tubercle. Therefore, the assessments of the lateral side contact seem to have no meaning in this stem. Additionally, we thought that stricter assessments were needed for the contact on the medial side. These are the reasons why we had not used the methods of Callaghan et al. There were no other reports on the press-fit or metaphyseal fit of the Anatomic stem.

We discuss the reason for the fact that the rate of good metaphyseal fit was not high. The data of the design of the Anatomic stem was obtained from normal femora of cadavers. Kaneuji et al. [13] studied the three-dimensional morphology of the femur on 113 hips with osteoarthritis and 36 normal hips in Japanese. In their study, the femoral canal was classified into three types, and the standard type accounted for 89% of the normal hips and only 42% of the hips with osteoarthritis. In our study, 117 hips out of 137 hips had been diagnosed as having osteoarthritis. The difference of femoral configuration between normal hip and osteoarthritis hip would be one of the reasons for poor metaphyseal fit. The undersized stem like Figure 4 also causes poor metaphyseal fit. However, no other stem was undersized like this case and showed loosening. Therefore, we think that the usage of undersized stem was not the main reason of poor metaphyseal fit.

Limitations of this study should be discussed. The metaphyseal fit was evaluated on AP radiographs. Three-dimensional analysis using CT scan would be more precise and is supposed to show lower rates of good. Since the mean followup of our study was 9.7 years, we cannot deny possible effects of metaphyseal fit on outcomes after longer followup. These points need further study.

## 5. Conclusions

The metaphyseal fit was good only in about 60% of cases, but 10-year survival rate of the stem was 99%. The biological fixation of the Anatomic Fiber Metal plus stem was stable at a mean followup of 9.7 years independently from metaphyseal fit.

## Figures and Tables

**Table 1:** The metaphyseal fit and outcomes of THA.

	Metaphyseal fixation		*P* value
	Good (*n* = 83)	Poor (*n* = 54)	
Followup (years)	9.4 ± 2.8	10.2 ± 3.0	0.07
JOA score after surgery	88 ± 11	86 ± 11	0.24
Stem fixation (B/U)	83/0	53/1	0.39
Radiolucent line in zone 4 (P/N)	64/19	42/12	0.93
Spot welds in zone 6 (P/N)	64/19	44/10	0.54
Osteolysis at medial of GT (P/N)	8/75	10/44	0.13
Stress shielding (grade I/ II)	82/1	51/3	0.14

B/U: bone ingrown/unstable, P/N: positive/negative, GT: greater trochanter.
